# Evaluating the impact of an integrated KIPP and positive psychology course on university students’ psychological well-being in China: a mixed-methods study

**DOI:** 10.3389/fpsyg.2025.1599216

**Published:** 2025-10-06

**Authors:** Tzu-Hsuan Liu, Huiwen Sun

**Affiliations:** ^1^School of Political Science and Public Administration, Huaqiao University, Quanzhou, China; ^2^School of Politics and Law, Public Administration, Northeast Normal University, Changchun, China

**Keywords:** KIPP, positive psychology, psychological well-being, mixed-methods study, university students

## Abstract

**Introduction:**

This study evaluated the impact of an integrated general education course—Building a Flourishing Life: Integrating KIPP and Positive Psychology—on the psychological well-being of university students in China. Drawing on the dual frameworks of the Knowledge is Power Program (KIPP) and the VIA character strengths model, the intervention aimed to promote happiness and reduce depression and anxiety.

**Methods:**

Using a mixed-methods approach, we collected pre- and post-intervention survey data (*N* = 116), performed fuzzy-set Qualitative Comparative Analysis (fsQCA) to identify configurational pathways, and applied directed content analysis to qualitative interview responses.

**Results:**

Quantitative results showed significant increases in happiness and decreases in depression and anxiety, which highlighted the additive and substitutive roles of strengths-based practices. Qualitative findings reinforced these outcomes. The fsQCA findings showed that no single module was necessary for the observed improvements in psychological well-being. Instead, multiple combinations of modules functioned synergistically.

**Conclusion:**

This study contributes to the growing literature on culturally responsive positive education and addresses recent critiques regarding the contextualization of psychological interventions in higher education.

## 1 Introduction

Mental health issues among Chinese students have become a significant concern in recent years. A meta-analysis of 113 studies has demonstrated a high prevalence of depression in this population, estimated at 28.4% (Gao et al., 2020). These challenges are often exacerbated by academic pressure, social expectations, and the complexities of transitioning to adulthood, highlighting the urgent need for effective interventions to enhance psychological well-being. In response, the integration of positive psychology into university curricula has emerged as a promising strategy. Globally, positive psychology principles have been increasingly applied in educational settings, described as a “fast-developing area of research” (Shankland and Rosset, 2017). Large-scale initiatives across diverse countries also underscore the international momentum behind positive education (Seligman and Adler, 2018).

Positive psychology, pioneered by Seligman and Peterson, emphasizes the cultivation of strengths, resilience, and overall well-being. As foundational figures in the field, Peterson and Seligman (2004) identified six core virtues—wisdom, courage, humanity, justice, temperance, and transcendence—operationalized through 24 culturally validated character strengths, which form the basis of the widely used Values in Action (VIA) Inventory of Strengths. This framework has been extensively integrated into educational settings, from early childhood programs to higher education curricula. A notable application of the VIA framework is the Knowledge is Power Program (KIPP), a U.S.-based public charter school network that emphasizes seven key character strengths—curiosity, gratitude, self-regulation, grit, social intelligence, optimism, and zest (Lester, 2024). Building on the principles of positive psychology, KIPP is a structured character education model that operationalizes these traits through embedded school practices, including the widely used Character Report Card (Duckworth, 2009). This model translates abstract constructs into observable behaviors and provides a practical framework for evaluating and supporting student growth.

The effectiveness of KIPP has been demonstrated in large-scale studies. A randomized controlled trial of over 2,000 students found that attending KIPP middle and high schools substantially increased college enrollment and graduation rates, closing gaps for Black and Hispanic students in the U.S. (Demers et al., 2023). Further evidence from the 2024 KIPP Impact Report showed that alumni graduate from college at three times the national average and benefit from mental health and career support (KIPP Foundation, 2024). International adaptations also show promise; for example, a Danish program inspired by KIPP and Seligman improved academic performance, motivation, and well-being among at-risk youth (Andersen et al., 2016). Relatedly, character strengths such as gratitude and optimism are consistently linked to greater happiness and lower distress across cultures (Nikoozadeh and Pirhayati, 2023; Brito and Soares, 2023; Martínez-Martí et al., 2020).

Despite these successes, few studies have examined how structured strength-based interventions can be adapted for the Chinese university context. Chinese students often experience high levels of perfectionism, parental pressure, and stigmatization surrounding mental health help-seeking (Liu et al., 2019; Ning et al., 2022). Lambert et al. (2023) emphasized the importance of culturally sensitive adaptations when applying Western psychological interventions. In response, we developed a course titled Building a Flourishing Life: Integrating KIPP and Positive Psychology, designed as a general education elective for Chinese undergraduates. The course represents a novel integration of the VIA framework, which provides the theoretical foundation, and the KIPP model, which offers a behaviorally grounded approach to character education. This dual-framework approach aims to create a developmentally appropriate, culturally relevant, and pedagogically feasible intervention to enhance student well-being. The course is structured into five thematic modules: Strengthening Relationships, which focuses on fostering empathy, kindness, and social intelligence; Discovering Strengths, Self-Regulation, and Balance, designed to help students identify their signature strengths and develop emotional regulation skills; Building Resilience, which equips students with strategies to overcome adversity and persevere through challenges; Finding Meaning and Joy (Transcendence), aimed at cultivating gratitude, hope, and a sense of purpose; and Integrating Strengths into Daily Life, which provides practical applications for using character strengths in academic, personal, and social contexts. By embedding KIPP’s seven key character strengths within the VIA framework, the course delivers a holistic and culturally grounded model for university student well-being.

While positive psychology interventions are increasingly used in educational contexts, the explicit integration of the KIPP character education model within a higher education course remains rare—especially in the Chinese cultural setting. Therefore, this study adopts a mixed-methods design to evaluate the impact of the integrated KIPP and Positive Psychology course on the psychological well-being of university students in China. Quantitative data were collected using standardized measures of happiness, depression, and anxiety, while qualitative insights were derived from in-depth student interviews. To rigorously assess the course’s effectiveness, three analytical strategies were employed: pre-post statistical analysis, fuzzy-set Qualitative Comparative Analysis (fsQCA), and a directed content analysis approach of qualitative data. To the best of our knowledge, no previous studies have employed a mixed-methods approach to assess the impact of such an integrated program in a Chinese university setting. This study not only contributes to the expanding body of literature on positive psychology’s application in higher education but also informs future initiatives aimed at addressing mental health challenges among university students in China. By bridging the gap between theory and practice, this research offers valuable insights into the potential of combining KIPP’s character education model with positive psychology to promote flourishing and resilience in university students.

The central research question guiding this study is:

RQ1: How does the course influence students’ psychological well-being?

Three hypotheses guide the quantitative analysis:

*H1a*: Students will report significantly higher happiness levels after completing the course.

*H1b*: Students will report significantly lower depression levels after completing the course.

*H1c*: Students will report significantly lower anxiety levels after completing the course.

To strengthen the coherence of the mixed-methods design, the fsQCA component is framed by the following exploratory research question:

RQ2: Which combinations of course modules are associated with higher levels of happiness and lower levels of depression and anxiety among students who completed the course?

The fsQCA, based on transformed quantitative data, aimed to uncover configurational patterns—that is, how different combinations of curricular modules are associated with psychological well-being. This exploratory approach is well suited for capturing complexity in educational contexts, where multiple pathways may be associated with similar mental health outcomes. It complements the variable-centered quantitative analysis by offering a system-level perspective on how course components interact to shape students’ well-being.

RQ3: How do students perceive the personal and psychological impact of the course, and what mechanisms do they identify as most influential in fostering well-being? This question guided the thematic analysis, which allowed us to triangulate findings and offer culturally contextualized interpretations of how students engaged with the course material and experienced its effects on their emotional lives.

In this study, the term “KIPP-PP integrated course” is used consistently to denote the intervention. This terminology highlights the deliberate integration of VIA-based positive psychology, which provides the theoretical foundations of character strengths and well-being, with the KIPP model, which offers practical and behaviorally grounded strategies for character development.

## 2 Materials and methods

### 2.1 Participants

The study employed a longitudinal design to track changes in participants’ psychological well-being throughout the duration of the KIPP-PP integrated course at a large university in Southeastern China. The pre-test questionnaire was administered during the first class session in September 2024, while the post-test was conducted during the final class session in December 2025. This data collection strategy yielded a high response rate of 97% across both survey administrations. All surveys were conducted in person during regular class sessions, which were part of a required general education course. No material incentives were offered for participation; however, students were informed that the surveys were part of the teaching evaluation process aimed at improving course quality and understanding student well-being. We acknowledge that this context may have introduced response bias due to students’ perceived obligation to comply with academic requirements. To match pre- and post-course responses while protecting participants’ privacy, a self-generated identification code system was used. Each student was instructed to create a unique alphanumeric code based on a combination of personal but non-identifying cues (e.g., the first letter of their first name + birth month + last digit of their student number). This code was used solely for data matching purposes and could not be linked back to the student’s identity by the researchers. All questionnaires were completed anonymously in person during class sessions, and no names, student numbers, or other identifying information were collected. Students were assured that their participation was voluntary, that their data would be kept confidential, and that their decision to participate or withdraw would have no impact on their course grades.

### 2.2 Intervention

The intervention consisted of the KIPP-PP integrated course. This 15-week course (100 min per week) was designed to synergize the theoretical principles of the VIA-based positive psychology framework with the practice-oriented structure of KIPP. The integration of these frameworks aimed to provide students with both the mindset and practical strategies to enhance psychological well-being. The course was organized around five thematic modules, each targeting one or more of the following core outcomes: increasing happiness, reducing depression, and reducing anxiety. These modules were not only theoretically informed but also grounded in applied activities such as gratitude journaling, character strength reflection, self-regulation training, and small-group discussions.

First, the Strengthening Relationships module focused on cultivating empathy, kindness, love, and social intelligence through various interpersonal activities such as group discussions, role-playing exercises, and collaborative projects. Students were encouraged to engage in exercises where they practiced active listening, shared personal experiences, and provided support to peers in building positive relationships. The module aimed to increase happiness by enhancing relational satisfaction and fostering emotional connections, especially beneficial for students struggling with social isolation or self-criticism. Additionally, the module worked to reduce depression and anxiety by offering emotional support, encouraging healthy communication, and helping students develop trust and meaningful connections with others.

Second, the Discovering Strengths, Self-Regulation, and Balance module emphasized the identification of personal signature strengths (e.g., perseverance, emotional regulation, self-awareness) and their mindful application in daily life. Activities included strength identification exercises, journaling prompts, self-reflection activities, and group discussions on how to utilize personal strengths. Students also participated in mindfulness meditation and cognitive reappraisal techniques, which helped them better manage stress and challenges. This module played a central role in reducing depression and anxiety by enhancing self-acceptance and providing students with tools for emotional regulation, while also contributing to increased happiness by fostering self-confidence, emotional growth, and a positive outlook on personal abilities.

Third, the Building Resilience module drew upon KIPP’s emphasis on grit and growth mindset, equipping students with coping strategies to face adversity and persevere through difficulties. Activities included reframing setbacks as opportunities, role-playing stressor responses, group problem-solving tasks, and personal storytelling where students shared challenges they had overcome. Students also engaged in goal-setting workshops to track progress and stay motivated in the face of challenges. This module aimed to reduce both depression and anxiety by building psychological flexibility and fostering a future-oriented, optimistic outlook, empowering students to better handle setbacks in both academic and personal contexts.

Fourth, the Finding Meaning and Joy (Transcendence) module, drawing from VIA’s transcendence virtues (e.g., hope, gratitude, appreciation of beauty), utilized gratitude journaling, mindfulness exercises, purpose-oriented reflection, and group activities such as appreciation circles where students shared moments of gratitude with peers. This module aimed to reduce anxiety and depression by cultivating inner calm and existential coherence, while simultaneously promoting increased happiness through the amplification of positive emotions like gratitude and joy. Students were also encouraged to engage in random acts of kindness and daily reflection on meaningful life moments, helping them appreciate the positive aspects of their lives.

Fifth, the Integrating Strengths into Daily Life module helped students translate learned character strengths into academic, social, and personal routines. For example, students practiced applying perseverance during exam preparation and emotional awareness in managing relationships. The module involved interactive workshops on time management, stress reduction techniques, and goal-setting with a focus on leveraging character strengths in real-life situations. This module supported all three psychological outcomes—happiness, depression, and anxiety—by reinforcing sustainable behavioral change and enhancing psychological self-efficacy. Students participated in peer support groups, where they shared their progress in applying character strengths and discussed challenges and successes. In sum, each module was designed to serve one or more of the psychological goals (increasing happiness, reducing depression, reducing anxiety) by engaging with empirically supported character strengths. The structure was both holistic and adaptable, reflecting the course’s dual commitment to theoretical integrity and cultural relevance within the Chinese higher education context. A detailed mapping of course modules, activities, and associated VIA/KIPP virtues is provided in Supplementary Table 1.

### 2.3 Measures

#### 2.3.1 Quantitative measures

Happiness was assessed using the Oxford Happiness Questionnaire (OHQ) (Hills and Argyle, 2002), a well-established self-report measure of subjective well-being. The OHQ comprises 28 items rated on a six-point Likert scale, with higher scores reflecting greater levels of happiness. The scale has demonstrated robust psychometric properties, including high internal consistency and strong construct validity across diverse populations. In this study, the OHQ was administered both before and after the course to evaluate changes in participants’ happiness levels. One item, “There is a gap between what I want to do and what I have done,” was removed not merely due to statistical indicators, but because multiple participants during the pilot testing and informal class feedback reported that its wording was vague and semantically ambiguous in the Chinese context. This item was considered culturally inappropriate and confusing, thus affecting the conceptual clarity of the scale. Empirically, the item showed a relatively weak factor loading and lower communality compared to other items. After removal, Cronbach’s *α* slightly increased (from 0.9411 to 0.9443), factor loadings became cleaner, and item–total correlations improved, with all remaining items exceeding accepted thresholds. These results suggest that the adapted OHQ retained strong reliability and improved construct validity in the Chinese university student sample.

Depression was measured using Tung’s Depression Inventory for college students, a self-report tool designed to assess depressive symptoms (Lin et al., 2008). The original scale consisted of 32 items, with responses ranging from “never or extremely rare – less than one day” (0) to “often or always – five to seven days” (3) per week. However, the item “I often space out” was perceived by students as unclear and not representative of depression symptoms in their everyday experience. Based on participant feedback and linguistic mismatch, we excluded the item to enhance the cultural appropriateness of the scale. Specifically, the item showed the lowest communality (0.438) and a weaker item–total correlation (*r* = 0.369) compared to the rest of the scale (most > 0.55). After removal, Cronbach’s *α* increased from 0.951 to 0.953, factor loadings became clearer, and item–total correlations strengthened, with all items demonstrating acceptable psychometric properties.

Anxiety was evaluated using the Burns Anxiety Inventory (Burns-A), a 33-item self-report scale designed to measure the severity of anxiety symptoms (Burns, 1993). The inventory is divided into three subscales: anxious feelings (6 items), anxious thoughts (11 items), and physical symptoms (16 items). The anxious feelings subscale assesses emotional states such as nervousness, excessive worry, and fear. The anxious thoughts subscale evaluates cognitive patterns associated with anxiety, including difficulties concentrating and fears of isolation, rejection, or losing control. The Physical Symptoms subscale captures somatic manifestations of anxiety, such as chest tightness, dizziness, muscle tension, and difficulty breathing. Participants rated each item based on the frequency of their experiences in recent days using a four-point Likert scale (0 = not at all, 3 = a lot). The anxiety inventory exhibited good internal consistency in this study, with a Cronbach’s *α* of 0.96.

#### 2.3.2 fsQCA measures

First, to explore the configurational pathways linking specific course components to psychological outcomes, we conducted fsQCA using calibrated post-intervention data. The fsQCA focused on identifying combinations of five thematic course modules—Strengthening Relationships, Discovering Strengths, Self-Regulation and Balance, Building Resilience, Finding Meaning and Joy, and Integrating Strengths into Daily Life—that were associated with high or low levels of happiness, depression, and anxiety.

Moreover, the three outcome variables were: Happiness (high vs. low): based on the post-test happiness scale, calibrated using the 75th percentile as the threshold for full membership (fuzzy score = 0.95), the 50th percentile for crossover (0.5), and the 25th percentile for full non-membership (0.05). Depression and Anxiety (low vs. high): reverse calibrated to reflect low depression and low anxiety as desirable outcomes, using similar percentile thresholds.

#### 2.3.3 Qualitative measures

For the qualitative component, semi-structured interviews were conducted with all 116 students who completed both the pre- and post-course questionnaires. Interviews took place immediately after the completion of the course during the final class session in December 2025, ensuring that participants could reflect on their experiences while the content and its impact were still fresh, thereby minimizing recall bias. Each interview lasted approximately 45 min and was conducted in a private meeting room on the university campus to provide a comfortable and confidential environment.

The interview guide was designed to explicitly probe the domains of happiness, depression, and anxiety to ensure alignment with the quantitative outcomes. Accordingly, a directed content analysis approach was employed. To ensure feasibility and quality, interviews were conducted by a team of ten trained research assistants, each with prior experience in qualitative interviewing. This structure enabled one-to-one interviews with all 116 participants within the available timeframe. Each assistant was assigned approximately 11 to 12 participants, allowing interviews to be completed in parallel while still ensuring depth and consistency. The research assistants followed a standardized semi-structured protocol, and all interviews lasted approximately 45 min. This team-based approach made it possible to achieve full coverage of the sample without compromising data quality. Interviews were audio-recorded with participant consent and later transcribed verbatim. Transcripts were anonymized, with only participant codes retained, and all qualitative data (audio files and transcripts) were stored securely on a password-protected university server accessible only to the research team. A codebook detailing the coding process and theme definitions was maintained separately from the data to preserve confidentiality.

Ethical approval for the qualitative component was obtained from the university’s Institutional Review Board (IRB). Participants provided informed consent, including agreement for audio recording and use of anonymized quotations in publications. For students aged 18 and above, only their individual consent was required. However, because the course included some first-year students who were 17 years old at the time of enrollment, parental or guardian consent was additionally obtained for these underage participants, as mandated by the university’s IRB guidelines. Raw data (e.g., transcripts, recordings) will not be publicly shared, but anonymized transcripts and the codebook may be made available to qualified researchers upon request, subject to ethical compliance.

Semi-structured interviews explored students’ subjective experiences of the course. The interview protocol included the following prompts: In what ways, if any, has your sense of happiness changed after taking this course? Can you describe any moments or activities in the course that helped you feel more emotionally uplifted? Have you noticed any changes in your feelings of anxiety, depression or happiness since participating in the course? Were there specific modules or practices that helped you cope better with negative emotions or daily challenges? How did the course influence your understanding or use of your personal strengths? Do you think the course content connected with your cultural background or personal values? Why or why not? These questions were anchored in the three core psychological targets of the course and guided a directed content analysis guided by pre-specified domains (happiness, depression, anxiety) of student reflections.

### 2.4 Data analysis

The present study adopted a mixed-methods approach to evaluate the psychological outcomes of the intervention, integrating quantitative analyses of pre-post changes with qualitative insights into students’ lived experiences. To ensure a comprehensive understanding of the intervention’s effects, a triangulated approach was used, integrating both quantitative and qualitative data. The quantitative results, derived from pre-post surveys, identified significant changes in students’ happiness, depression, and anxiety. The fsQCA and qualitative interviews, conducted after the quantitative data collection, provided a deeper understanding of these changes by capturing students’ personal reflections and perceptions of the course. The qualitative phase used directed content analysis to elaborate how and why changes might have occurred, treating interviews as complementary evidence aligned to the three predefined outcomes rather than as fully inductive theme generation. We also coded and reported disconfirming evidence (neutral, mixed, and critical) to reduce confirmation bias. Findings are framed as illustrative accounts within these domains. This integration of both data types helped contextualize and explain the quantitative findings, offering a fuller picture of the intervention’s impact on students’ well-being.

The data collection was conducted sequentially. First, quantitative data were collected through pre- and post-test surveys, assessing happiness, depression, and anxiety before and after the course. These data were analyzed using paired-sample t-tests. Following the quantitative analysis, fsQCA and qualitative interviews were conducted with all participants to capture their reflections on the course. The interviews were analyzed using thematic analysis, allowing us to identify key themes related to the students’ experiences and perceived outcomes. By conducting qualitative analysis after the quantitative analysis, we were able to provide further insight into the patterns identified in the quantitative phase, enriching our understanding of how specific course components influenced students’ psychological well-being.

The qualitative component of this study was exploratory in nature, aimed at gaining in-depth insights into students’ lived experiences, perceptions, and reflections on the course. This qualitative approach served to complement the quantitative data, providing a more nuanced understanding of how the intervention influenced students’ psychological well-being. The qualitative interviews helped to explain the patterns observed in the quantitative analysis and provided additional context regarding the mechanisms through which the course affected students’ happiness, depression, and anxiety.

#### 2.4.1 Quantitative analysis

Paired-sample t-tests were conducted to assess within-subject changes in psychological well-being—namely, happiness, depression, and anxiety—across the intervention period. While these analyses provided estimates of average treatment effects at the group level, they did not capture the configurational complexity by which distinct combinations of course modules might differentially influence outcomes.

#### 2.4.2 fsQCA analysis

To complement the variable-centered approach of paired-sample t-tests, we employed fsQCA to identify multiple, equifinal pathways through which course modules influenced students’ psychological outcomes. Unlike regression models, which assume linear and additive effects, fsQCA enables the exploration of configurational complexity by examining how different combinations of conditions are associated with similar results (Fiss, 2011; Greckhamer et al., 2018).

Although our sample size (*N* = 116) may appear modest, it is well within the suitable range for fsQCA applications. While the method was originally developed for small and intermediate samples (<50), recent systematic reviews have demonstrated its application across a wide spectrum of sample sizes, including studies with several thousand cases, underscoring its methodological flexibility (Geremew et al., 2024). Importantly, fsQCA has been increasingly applied in educational research to uncover multiple equifinal pathways to outcomes, such as in studies of classroom silence (Chen N. C. et al., 2025) and teachers’ adoption of digital learning tools (Chen J. et al., 2025). These precedents reinforce the appropriateness of fsQCA for our evaluation of a multi-component university course.

In our mixed-methods evaluation of the KIPP-PP integrated course—which comprises five interactive modules—fsQCA was particularly valuable for capturing the nuanced ways in which different module combinations fostered well-being across student subgroups. This configurational insight complements the aggregate-level changes identified through t-tests and enriches understanding of how course components collectively are associated with psychological outcomes. Notably, while many studies use fsQCA as a stand-alone method, our design integrates fsQCA alongside pre–post quantitative analysis and qualitative thematic analysis. This triangulated approach strengthens validity by linking statistical evidence of improvement, configurational pathways of module engagement, and students’ lived experiences.

The fsQCA was conducted on calibrated post-test data. Condition variables reflected participation in each of the five modules (coded as 1 = full engagement, 0 = limited or no engagement) based on both instructor attendance records and student self-reports. Outcome variables—happiness, depression, and anxiety—were transformed into fuzzy sets using the direct calibration method (Ragin, 2008), with anchors at 0.95 (full membership), 0.50 (crossover), and 0.05 (full non-membership). To assess robustness, sensitivity analyses were conducted by applying alternative calibration thresholds (0.90 / 0.45 / 0.10) alongside the original (0.95 / 0.50 / 0.05). Depression and anxiety were reverse-calibrated so that higher fuzzy scores reflected more desirable psychological states (i.e., lower symptoms). This system-level approach revealed multiple equifinal pathways that would remain invisible in variable-centered analyses. By embedding fsQCA within a mixed-methods framework, our study leveraged its unique strengths to capture the heterogeneous ways students benefited from the KIPP-PP integrated course.

#### 2.4.3 Qualitative analysis

We analyzed transcripts using a directed content analysis strategy (Hsieh and Shannon, 2005). First-cycle coding applied *a priori* categories (increase in happiness, reduction in depression, reduction in anxiety) derived from the interview guide and study hypotheses. Second-cycle coding identified mechanism-oriented subcodes (e.g., emotional regulation, gratitude practices, meaning-making, relationship repair) and countervailing patterns (neutral, mixed, and critical responses). Two independent coders worked blind to each other’s first-cycle codes; discrepancies were adjudicated, achieving Cohen’s *κ* = 0.85. While our categories were deductively anchored, subcodes were developed to nuance each domain. The final report illustrates student accounts within the three predefined domains (happiness, depression, anxiety) and documents boundary conditions, rather than presenting inductive theme generation.

The interviews were conducted in Chinese, as the study was conducted at a university in China, and all participants were Chinese-speaking. After the interviews, the transcripts were translated into English by a bilingual researcher proficient in both Chinese and English to ensure accuracy and consistency. Moreover, a bilingual coder reviewed the final English translations and the original Chinese transcripts to ensure consistency and preserve intended meaning. Audio recordings were transcribed verbatim and carefully reviewed for accuracy. Moreover, two independent coders conducted the analysis. Although the first author was the course instructor, bias was minimized by training both coders on the VIA framework and KIPP model, ensuring they worked independently during coding, and blinding the first author to the second coder’s initial codes. Coders were also blinded to each other’s initial codes, with discrepancies resolved through discussion, achieving intercoder reliability (Cohen’s *κ* = 0.85). Member checking with 20 participants confirmed the accuracy of themes. To further mitigate bias, the first author kept a reflexive journal, coders maintained awareness of positionality, and peer review and member checking ensured findings were grounded in participants’ voices rather than researcher expectations.

## 3 Results

The final sample consisted of 116 undergraduate students. Participants were from diverse academic disciplines, including Business (*n* = 30), Fine Arts (*n* = 25), Communication (*n* = 24), Physical Education (*n* = 20), Politics and Public Administration (*n* = 10), and Engineering (*n* = 7), reflecting a broad representation across the university (see Table 1). The mean age of participants was 19.8 years (SD = 1.2), ranging from 17 to 22. In terms of university year level, students came from all four academic years, with an average level of 2.32 (SD = 1.07). In terms of year of study, 32 students were in Year 1, 28 in Year 2, 31 in Year 3, and 25 in Year 4. As for gender distribution, of the 116 participants, 68 were male and 48 were female.

**Table 1:** Distribution of student participants (*N* = 116) across academic disciplines.

Discipline	*n*
Business School	30
School of Fine Arts	25
School of Politics and Public Administration	10
School of Physical Education	20
School of Communication	24
School of Engineering	7

Reported values indicate the number of students from each school (e.g., Business School, Fine Arts, Politics and Public Administration, Physical Education, Communication, Engineering).

To address mental health baseline status and potential course effects, pre- and post-test data were collected using standardized self-report measures of happiness, depression, and anxiety. Results indicated a modest improvement in subjective well-being, with happiness scores increasing from a mean of 3.97 (SD = 0.75) to 4.07 (SD = 0.74). More notably, depression scores decreased from 21.14 (SD = 15.09) to 16.28 (SD = 11.74), and anxiety scores dropped from 20.67 (SD = 16.78) to 15.63 (SD = 13.32), suggesting reductions in negative affective symptoms (see Table 2).

**Table 2:** Descriptive statistics of student participants in the quantitative phase (*N* = 116).

Variable	Mean	SD	Min	Max
Gender	0.41	0.495	0	1
University Year Levels	2.32	1.068	1	4
Age	19.8	1.2	17	22
Happiness Pre-test	3.97	0.747	2	6
Happiness Post-test	4.07	0.735	2	6
Depression Pre-test	21.14	15.089	0	67
Depression Post-test	16.28	11.743	0	59
Anxiety Pre-test	20.67	16.779	0	76
Anxiety Post-test	15.63	13.315	0	61

Reported values include means, SD, and observed ranges (minimum–maximum) for demographic variables (gender, age, university year level) and psychological measures (happiness, depression, anxiety) assessed at both pre-test and post-test. Higher scores on the Oxford Happiness Questionnaire (OHQ; range: 1–6) reflect greater happiness, whereas higher scores on the Oxford Depression Questionnaire (range: 0–93) and Oxford Anxiety Questionnaire (range: 0–99) reflect greater symptom severity.

### 3.1 Paired sample correlations

The correlations between pre- and post-course scores were analyzed to assess the consistency of participants’ responses over time. The results revealed strong and statistically significant associations for all three variables. For happiness, the correlation coefficient was *r* = 0.821 ( *p* < 0.001), suggesting a high degree of consistency in participants’ self-reported happiness levels before and after the course. Similarly, for depression, the correlation was *r* = 0.741 ( *p* < 0.001), demonstrating a strong relationship between pre- and post-course scores. Anxiety also showed a robust correlation, with *r* = 0.784 ( *p* < 0.001) (Table 3). These findings suggest that participants’ responses were highly stable across the two time points, reinforcing the reliability of the observed changes and providing confidence in the validity of the effects.

**Table 3:** Paired-samples correlations (*N* = 116) for happiness, depression, and anxiety between pre-test and post-test scores.

Variable	*N*	Correlation	Sig.(two-tail)
Happiness	116	0.821	*p* < 0.001
Depression	116	0.741	*p* < 0.001
Anxiety	116	0.784	*p* < 0.001

Correlation coefficients and significance levels (two-tailed) are presented. All correlations were statistically significant at p < 0.001.

### 3.2 Paired-sample t-tests

Paired-sample t-tests were conducted to evaluate whether the observed changes in happiness, depression, and anxiety were statistically significant. To address the issue of inflated Type I error due to multiple comparisons, Bonferroni correction was applied (adjusted *α* = 0.05/3 = 0.017). The results demonstrated statistically significant improvements across all three psychological outcomes, with accompanying effect sizes to reflect practical significance (Table 4).

**Table 4:** Pairwise comparisons of pre-test and post-test scores for happiness, depression, and anxiety (*N* = 116).

Variable	Diff.(Pre-test minus post-test)	SD	SEM	95% CI		*t*	df	Sig.(two-tail)
				Lower	Upper			
Happiness	−0.11	0.44	0.04	−0.19	−0.03	−2.65	115	*p* = 0.009
Depression	4.86	10.15	0.94	3.00	6.73	5.16	115	*p* < 0.001
Anxiety	5.04	10.42	0.97	3.13	6.96	5.21	115	*p* < 0.001

Reported values include mean differences, standard deviations (SD), standard errors of the mean (SEM), 95% confidence intervals, t statistics, degrees of freedom (df), and two-tailed significance levels. Negative mean differences indicate positive change (e.g., reduction in symptoms), while positive mean differences indicate negative change.

**Figure 1:**
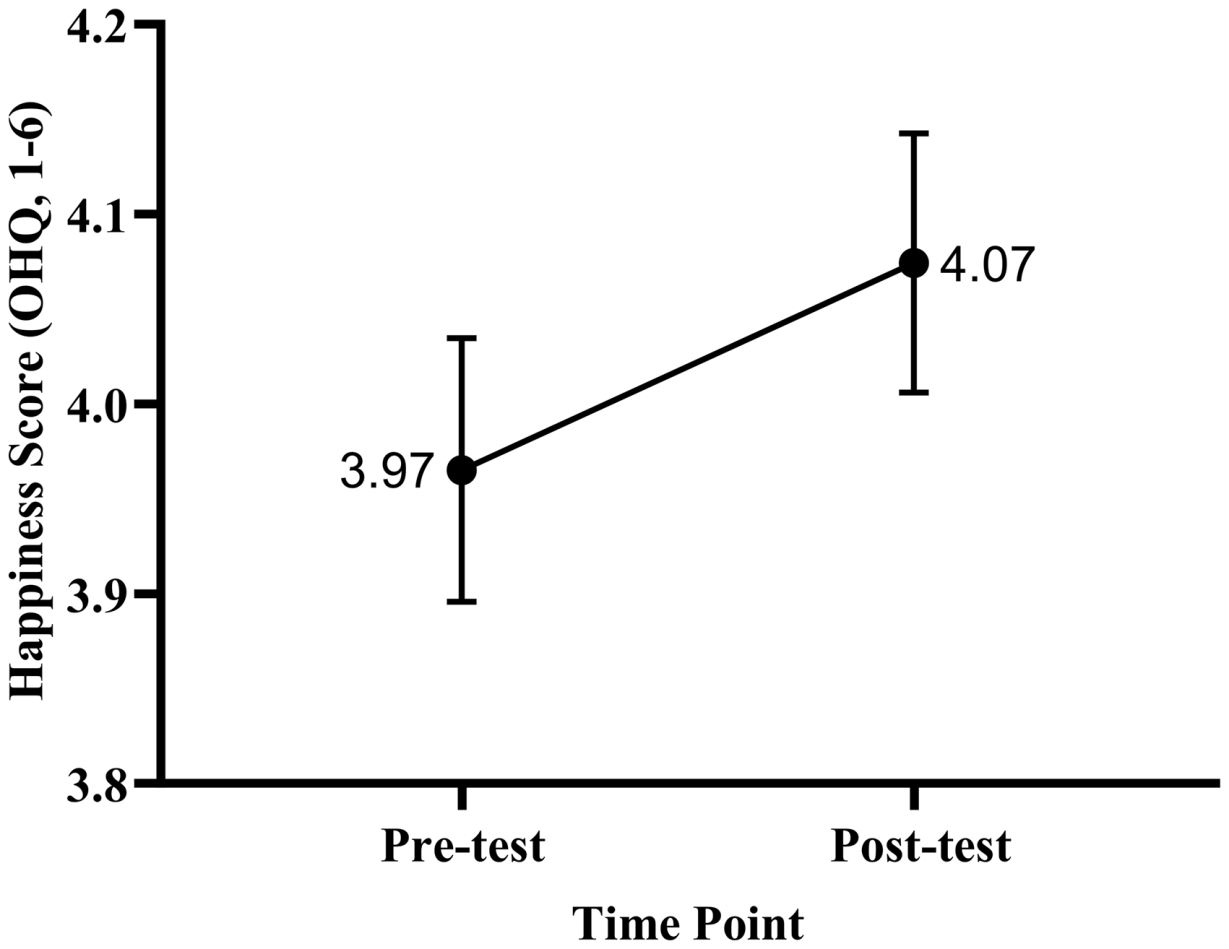
Line plot of mean happiness scores before and after the course (*N* = 116). Happiness was measured using the Oxford Happiness Questionnaire (OHQ; range: 1–6). Error bars represent standard errors of the mean (SEM). Numbers indicate mean scores at each time point (Pre-test = 3.97, Post-test = 4.07). A paired-samples t-test indicated a statistically significant increase in happiness (*t* (115) = −2.65, *p* = 0.009).

**Figure 2:**
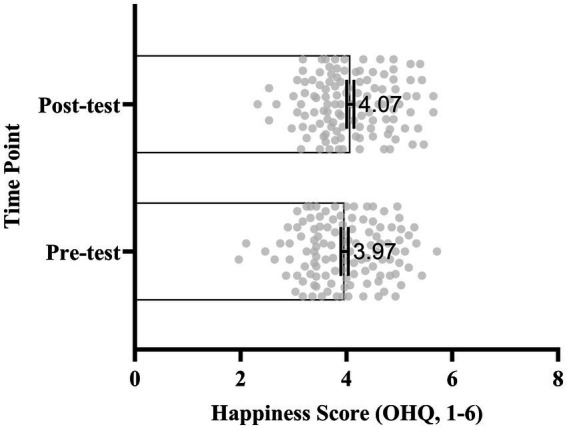
Scatter and box plot of individual happiness scores (*N* = 116) at pre- and post-test. Vertical axis: time point (pre-test vs. post-test). Horizontal axis: OHQ scores (range: 1–6). Scatter points represent individual students (jittered for clarity), boxes indicate interquartile range, whiskers show full range, and inner lines represent the mean. Mean scores: Pre-test = 3.97, Post-test = 4.07.

**Figure 3:**
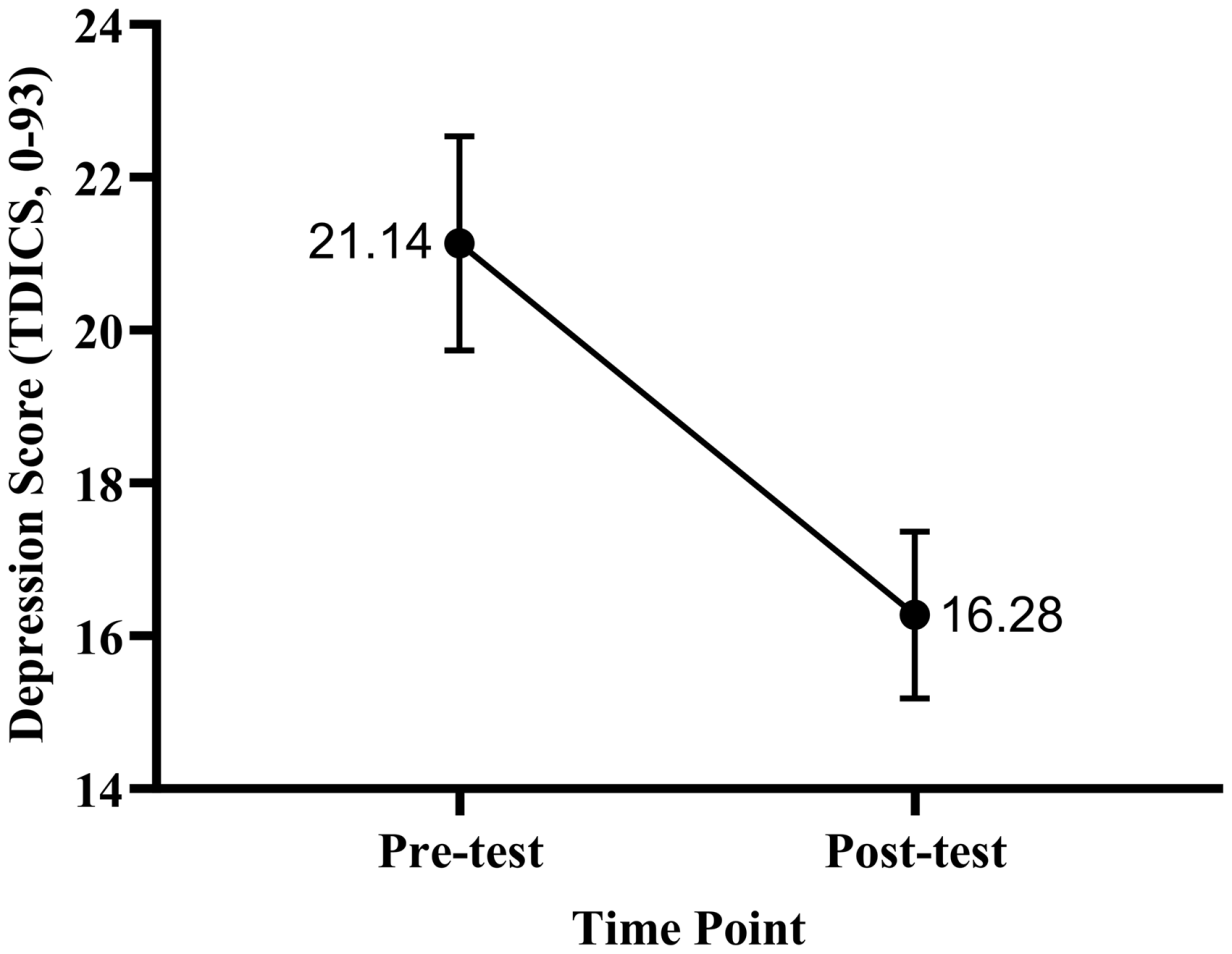
Change in mean depression scores before and after the course (*N* = 116). Depression was measured using the Oxford Depression Questionnaire (range: 0–93). Error bars represent SEM. Numbers indicate mean scores (Pre-test = 21.14, Post-test = 16.28). A paired-samples t-test revealed a significant reduction in depression following the course (*t* (115) = 5.16, *p* < 0.001).

**Figure 4:**
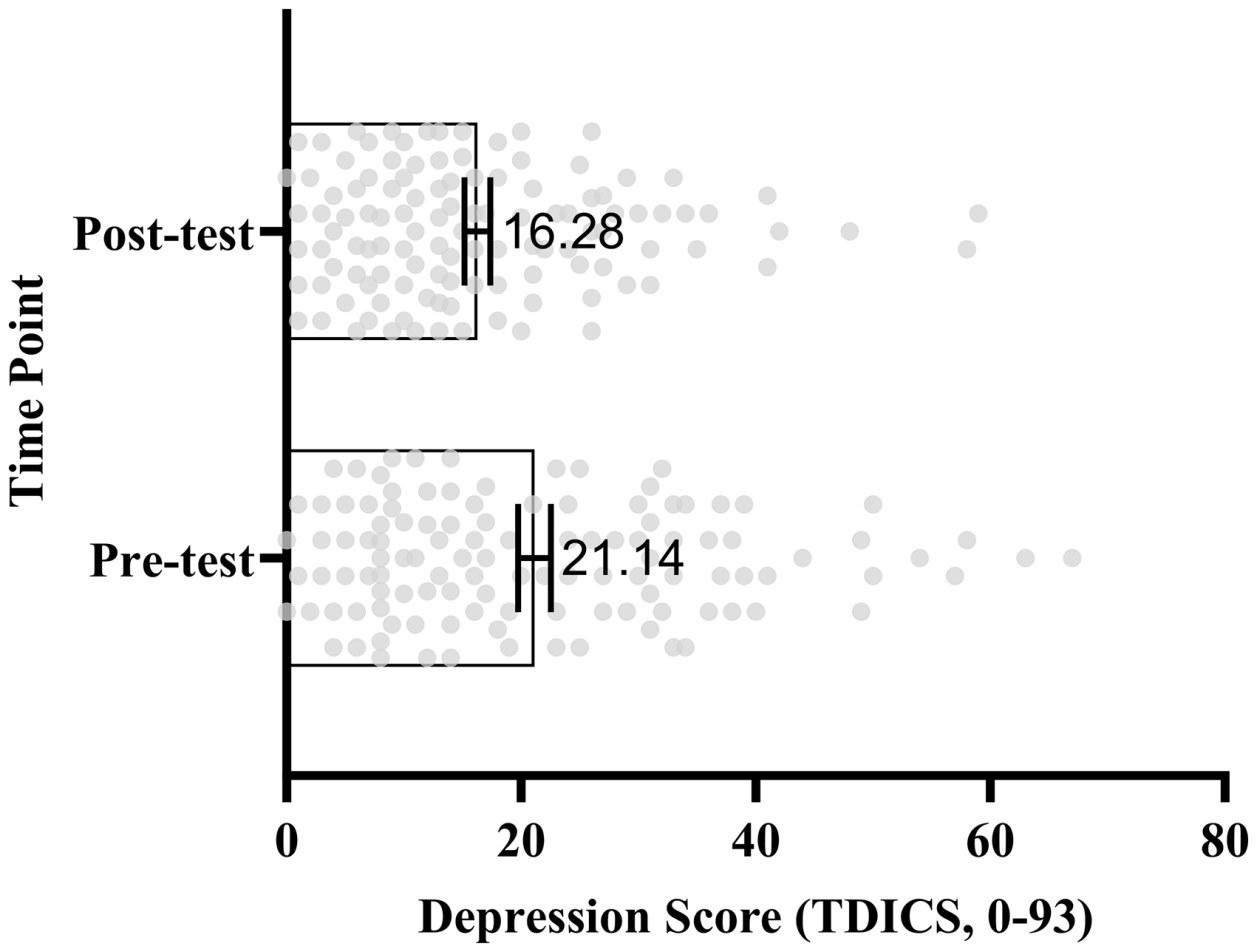
Scatter and box plot of individual depression scores (*N* = 116) at pre- and post-test. Vertical axis: time point (pre-test vs. post-test). Horizontal axis: Depression scores (range: 0–93). Scatter points represent individual students (jittered for clarity), boxes indicate interquartile range, whiskers show full range, and inner lines represent the mean. Mean scores: Pre-test = 21.14, Post-test = 16.28.

For happiness, the mean difference was −0.11 (SD = 0.44), *t* (115) = −2.65, *p* = 0.009 (Bonferroni-corrected *p* = 0.027), indicating a small but significant increase in subjective happiness following the course. The effect size was small (Cohen’s d = 0.25), suggesting that although the change was statistically reliable, its practical magnitude was modest (Figures 1, 2). For depression, the analysis revealed a mean reduction of 4.86 points (SD = 10.15), *t* (115) = 5.16, *p* < 0.001 (Bonferroni-corrected *p* < 0.001), with a moderate effect size (Cohen’s d = 0.48). This finding suggests a significant decrease in depressive symptoms among participants (Figures 3, 4).

**Figure 5:**
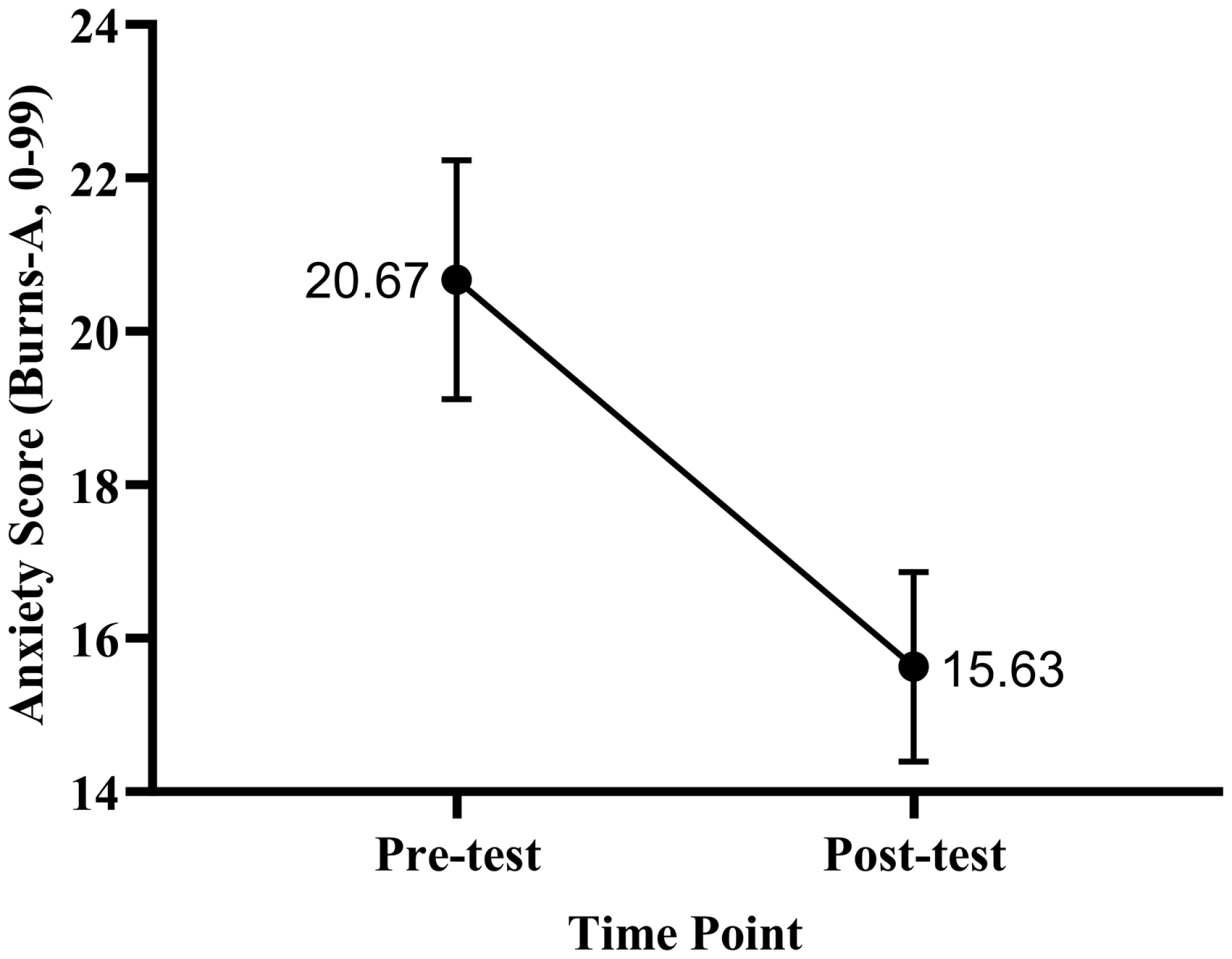
Change in mean anxiety scores before and after the course (*N* = 116). Anxiety was measured using the Oxford Anxiety Questionnaire (range: 0–99). Error bars represent SEM. Numbers indicate mean scores (Pre-test = 20.67, Post-test = 15.63). A paired-samples t-test revealed a significant reduction in anxiety following the course (*t* (115) = 5.04, *p* < 0.001).

**Figure 6:**
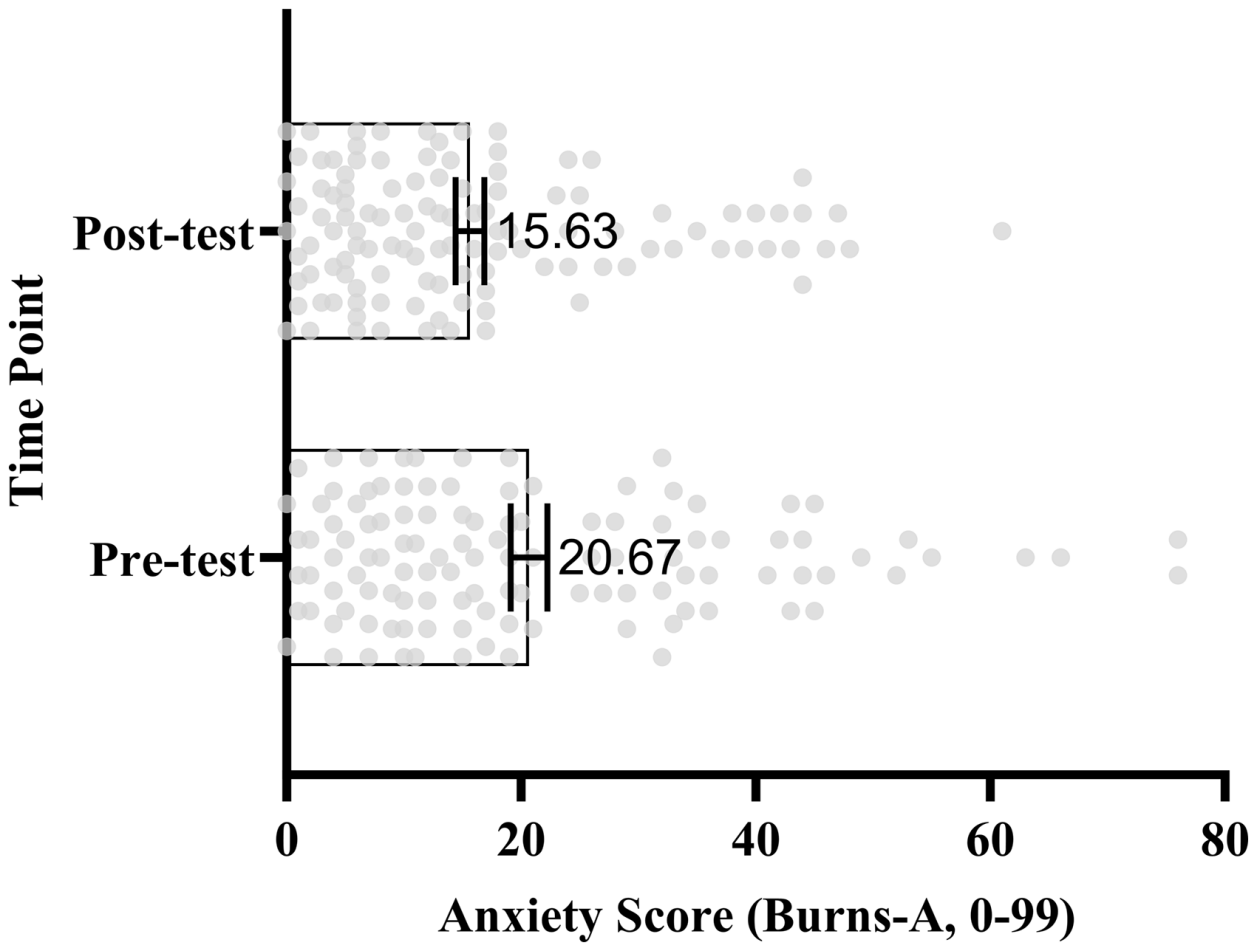
Scatter and box plot of individual anxiety scores (*N* = 116) at pre- and post-test. Vertical axis: time point (pre-test vs. post-test). Horizontal axis: Anxiety scores (range: 0–99). Scatter points represent individual students (jittered for clarity), boxes indicate interquartile range, whiskers show full range, and inner lines represent the mean. Mean scores: Pre-test = 20.67, Post-test = 15.63.

For anxiety, the mean reduction was 5.04 points (SD = 10.42), *t* (115) = 5.21, *p* < 0.001 (Bonferroni-corrected *p* < 0.001), accompanied by a moderate effect size (Cohen’s d = 0.48). These results suggest a significant decrease in anxiety symptoms post-course (Figures 5, 6).

### 3.3 Results from the fsQCA

The fsQCA analysis examined how different combinations of course modules were associated with students’ psychological outcomes. No single module was observed to function as a necessary condition for happiness, depression, or anxiety, implying that well-being outcomes were associated with the combined influence of multiple components rather than any single dominant factor.

First, regarding happiness, two distinct pathways were associated with higher or lower happiness. Students who engaged in both Discovering Strengths, Self-Regulation and Balance and Building Resilience reported greater happiness, suggesting that self-regulatory and resilience-based skills were linked to positive affect. In contrast, students who primarily participated in Strengthening Relationships and Integrating Strengths into Daily Life without sufficient support from self-regulation and resilience modules tended to report lower happiness. Table 5 displays configurations that are associated with happiness.

**Table 5:** Configurations that are associated with happiness.

Configuration	Solution high	Solution low
	1	2
Strengthening Relationships	□	●
Discovering Strengths, Self-Regulation and Balance	●	○
Building Resilience	●	○
Finding Meaning and Joy (Transcendence)	○	○
Integrating Strengths into Daily Life		●
Consistency	0.85	0.88
Raw coverage	0.10	0.02
Unique coverage	0.10	0.02
Solution consistency	0.85	0.88
Solution coverage	0.10	0.02

● = presence of a core condition; ■ = presence of a peripheral condition; ○ = negation of a core condition; □ = negation of a peripheral condition; Blank spaces indicate that a condition does not matter in a configuration. Consistency, raw coverage, unique coverage, and solution coverage are reported.

Second, as for depression, four different pathways were linked to reduced depression. Most prominently, configurations combining Strengthening Relationships, Building Resilience, and Finding Meaning and Joy were associated with lower depressive symptoms, highlighting the importance of social connection, coping strategies, and meaning-making. Alternative configurations also showed that self-regulation and daily strength application could buffer against depression even when interpersonal modules were less central. Table 6 displays configurations that are associated with depression.

**Table 6:** Configurations that are associated with depression.

Configuration	Solution high			Solution low
	1	2	3	4
Strengthening Relationships	●	□	■	■
Discovering Strengths, Self-Regulation and Balance	○	●	○	●
Building Resilience	●	●	○	○
Finding Meaning and Joy (Transcendence)	●	○	●	●
Integrating Strengths into Daily Life	○	●	●	●
Consistency	0.82	0.80	0.80	0.86
Raw coverage	0.03	0.04	0.04	0.02
Unique coverage	0.03	0.04	0.04	0.02
Solution consistency	0.80	0.86		
Solution coverage	0.10	0.02		

● = presence of a core condition; ■ = presence of a peripheral condition; ○ = negation of a core condition; □ = negation of a peripheral condition; Blank spaces indicate that a condition does not matter in a configuration. Multiple solution pathways are reported, each with consistency, coverage, and solution statistics.

Third, regarding anxiety, two stable pathways were associated with reduced anxiety. In some cases, Integrating Strengths into Daily Life alone was sufficient to align with lower anxiety, underscoring the value of applying character strengths in everyday routines. Another pathway involved Strengthening Relationships, Building Resilience, and Finding Meaning and Joy, which together provided relational, emotional, and existential resources for managing stress. Table 7 displays configurations that are associated with depression.

**Table 7:** Configurations that are associated with anxiety.

Configuration	Solution high	
	1	2
Strengthening Relationships	○	●
Discovering Strengths, Self-Regulation and Balance	□	○
Building Resilience	○	●
Finding Meaning and Joy (Transcendence)	□	●
Integrating Strengths into Daily Life	●	○
Consistency	0.95	0.95
Raw coverage	0.02	0.03
Unique coverage	0.02	0.03
Solution consistency	0.95	
Solution coverage	0.04	

● = presence of a core condition; ■ = presence of a peripheral condition; ○ = negation of a core condition; □ = negation of a peripheral condition; Blank spaces indicate that a condition does not matter in a configuration. Two solution pathways are presented, with coverage and consistency values.

Overall, these results indicate that improvements in well-being were associated with multiple, equifinal configurations of course modules, consistent with the configurational logic of fsQCA. Detailed calibration thresholds, and necessity analyses are reported in the Supplementary Tables S2–S5.

Besides, sensitivity analyses using stricter calibration thresholds (0.90/0.45/0.10) produced results that were consistent with the original models, with only minor variations for depression. Detailed statistics are reported in the Supplementary Tables S6–S9.

### 3.4 Qualitative results

In line with our directed content analysis approach, the results are organized into three themes corresponding to the predefined outcome domains gained from the integrated KIPP and Positive Psychology course: reduction in depression, reduction in anxiety, and increase in happiness. These outcomes were attributed to specific combinations of course modules and were further aligned with corresponding KIPP/VIA character virtues. Table 8 displays the qualitative results. Illustrative quotes and virtue mapping are presented thematically below.

**Table 8:** Summary of qualitative themes derived from student interviews (*N* = 116).

Theme	Description (with student quotes)	Course modules involved	Corresponding KIPP/VIA virtues
Increase Happiness	Strengthening Relationships module and Integrating Strengths into Daily Life module increased my happiness by improving my self-acceptance and positive mindset. After this course, I learned to embrace both my strengths and weaknesses, which made me feel more confident and happier. (P16)	Strengthening Relationships module + Integrating Strengths into Daily Life module	Self-acceptance, Optimism, Social Intelligence, Gratitude, Love, Perspective, Zest
Increase Happiness	Discovering Strengths, Self-Regulation and Balance module, and Building Resilience module increased my happiness by offering a clear framework for self-development and emotional regulation. The course gave me a structured path for personal growth, emotional balance, and stronger relationships. It helped me live a healthier, happier life. (P7)	Discovering Strengths, Self-Regulation and Balance module + Building Resilience module	Self-Regulation, Growth, Zest, Hope, Perspective, Optimism, Social Intelligence, Self-Acceptance, Cognitive Reappraisal, Optimism, Gratitude, Emotional Awareness, Self-Compassion
	By learning to reframe challenges as growth opportunities, I became more confident and positive, which significantly increased my happiness. Now I face difficulties with a better mindset and improved relationships—I feel much happier. (P34)		
	These modules increased my happiness by enhancing my self-recognition. The course helped me realize I’m not as bad as I thought—I gained more self-acceptance and happiness. (P46)		
	Practicing gratitude and accepting emotions helped increase my happiness. Writing down things I’m grateful for brought me joyful moments. I’ve learned to face anxiety, tension, and sadness more calmly, which improved my happiness. (P78)		
Reduce Depression	The combined modules of Strengthening Relationship, Finding Meaning and Joy (Transcendence), and Integrating Strengths into Daily Life helped reduce my depression by teaching me to view relationships with optimism and empathy. It brought positive energy to my relationship and gave me a goal to pursue in life.(P24)	Strengthening Relationship module + Finding Meaning and Joy (Transcendence) module + Integrating Strengths into Daily Life module	Love, Perspective, Self-regulation, Hope, Social Intelligence, Emotional Strength, Courage, Self-Awareness Grit, Optimism
Reduce Depression	The combination of “Strengthening Relationship” module, Discovering Strengths, Self-Regulation and Balance, Finding Meaning and Joy (Transcendence), Integrating Strengths into Daily Life module helped me reduce depression by teaching me to persevere through life’s challenges. I realized that life is not always joyful, but overcoming difficulties makes us stronger. As long as we do not give up, there’s always light ahead. (P9)	Strengthening Relationship module + Discovering Strengths, Self-Regulation and Balance module + Finding Meaning and Joy (Transcendence) module + Integrating Strengths into Daily Life” module	Grit, Hope, Self-Regulation, Perseverance, Optimism
Reduce Depression	These modules (Discovering Strengths, Self-Regulation and Balance module, Building Resilience module, and Integrating Strengths into Daily Life module) helped me reduce depression by strengthening self-awareness and emotional balance. They taught me how to face difficulties and remain strong. Overcoming hardship gave me hope and reduced my depressive feelings. (P23)	Discovering Strengths, Self-Regulation and Balance module + Building Resilience module + Integrating Strengths into Daily Life module	Gratitude, Self-Regulation, Perseverance, Optimism, Zest, Hope, Curiosity
Reduce Depression	These modules (Strengthening Relationship module, Building Resilience module, and Finding Meaning and Joymodule) helped reduce my depression by fostering self-acceptance and emotional stability. After taking this course, I feel calmer and more resilient. I’ve learned not to let others’ opinions hurt me—only by loving and affirming myself can I feel truly confident. It has significantly reduced my depression. (P101)	Strengthening Relationship module + Building Resilience module + Finding Meaning and Joy (Transcendence) module	Self-Regulation, Authenticity, Social Intelligence, Self-Respect, Hope, Zest, Perspective, Optimism, Gratitude, Self-Awareness
Reduce Depression	These modules improved my mindset and helped reduce my depression by encouraging optimism, self-knowledge, and better relationships. This course changed how I deal with stress. I now see setbacks as growth opportunities, feel more confident in my abilities, and resolve conflicts more calmly. All of this helped reduce my depression. (P64)	Strengthening Relationship module + Building Resilience module + Finding Meaning and Joy (Transcendence) module	Self-Regulation, Authenticity, Social Intelligence, Self-Respect, Hope, Zest, Perspective, Optimism, Gratitude, Self-Awareness
Reduce Anxiety	These modules helped reduce my anxiety by enhancing emotional stability and self-acceptance. After these classes, I feel calmer and more grounded. I learned not to care too much about others’ judgment, and to protect my mental space. Loving myself has helped me reduce anxiety. (P101)	Strengthening Relationship module + Building Resilience module + Finding Meaning and Joy (Transcendence) module	Self-Respect, Self-Regulation, Social Intelligence, Gratitude, Hope, Zest, Perspective, Optimism, Self-Awareness
	Practicing gratitude through these modules helped me shift focus from worries to positive feelings, effectively reducing anxiety. The greatest help was learning to be grateful. It not only lifted my mood but clearly reduced my anxiety.(P68)		
	Sharing experiences with classmates during the Strengthening Relationships module made me feel supported and less isolated. Knowing that others had similar struggles reduced my anxiety and gave me more confidence in facing challenges.(P73)		
Reduce Anxiety	These modules helped me develop a better mindset and stronger interpersonal skills, which reduced my anxiety in daily life. This course gave me a healthier outlook. I worry less when facing setbacks and understand myself better. I’m more patient with others, and my life feels more peaceful—my anxiety has definitely reduced.” (P65)	Strengthening Relationship module + Building Resilience module + Finding Meaning and Joy (Transcendence) module	Self-Respect, Self-Regulation, Social Intelligence, Gratitude, Hope, Zest, Perspective, Optimism, Self-Awareness
	“The relaxation and breathing practices we learned in class helped me manage stress more calmly. When I feel anxious before exams, I now use these techniques to steady my emotions and focus better.” (P42)		

Themes include representative student quotes, corresponding course modules involved, and aligned KIPP/VIA character strengths (e.g., love, hope, perseverance, grit, self-regulation).

#### 3.4.1 Theme 1: increase in happiness

Students reported increased happiness through enhanced self-recognition, emotional regulation, gratitude, and relationship-building. This directly addresses RQ1 and supports H1a. The Discovering Strengths, Self-Regulation and Balance and Building Resilience modules were frequently cited. *“I became more confident and positive, which significantly increased my happiness.”* (P34). This reflects how emotional regulation within the Self-Regulation and Balance module fostered confidence and happiness.

Additionally, students described how Strengthening Relationships and Integrating Strengths into Daily Life modules supported happiness through improved romantic relationships, savoring positive memories, and balanced self-appraisal. *“I learned to embrace both my strengths and weaknesses, which made me feel more confident and happier.”* (P16). Overall, 40% of participants highlighted emotional regulation as a core strategy, and 30% emphasized self-acceptance and relationship-building as key to improved emotional well-being.

#### 3.4.2 Theme 2: reduction in depression

Student accounts illustrated that the integrated modules helped alleviate depressive symptoms by fostering emotional regulation, cognitive reframing, and self-awareness. This directly addresses RQ1: How does the course influence students’ psychological well-being? Specifically, it supports H1b.

For example, the combination of the Strengthening Relationships, Finding Meaning and Joy (Transcendence), and Integrating Strengths into Daily Life modules was especially impactful. *“The course helped reduce my depression by teaching me to view relationships with optimism and empathy. It brought positive energy to my relationship and gave me a goal to pursue in life.”* (P24).

Another group of students emphasized how perseverance and meaning-making played a central role in mitigating depressive thoughts. They particularly referenced the Discovering Strengths, Self-Regulation and Balance, and Finding Meaning and Joy modules. *“I realized that life is not always joyful, but overcoming difficulties makes us stronger. As long as we do not give up, there’s always light ahead.”* (P9). In total, 40% of students mentioned improved relationships as reducing depression, and 45% highlighted meaning-making as crucial for mitigating depressive thoughts.

#### 3.4.3 Theme 3: reduction in anxiety

Students reported that the Strengthening Relationships, Building Resilience, and Finding Meaning and Joy (Transcendence) modules helped them reduce anxiety by promoting self-acceptance, emotional stability, and relational understanding. This directly responds to RQ1 regarding the course’s effect on psychological well-being, particularly H1c. *“After taking this course, I feel calmer and more grounded. I’ve learned not to let others’ opinions hurt me—only by loving and affirming myself can I feel truly confident. It has significantly reduced my depression and anxiety.”* (P101).

Another student described gratitude as a tool for redirecting emotional attention away from worry: *“Practicing gratitude helped me shift focus from worries to positive feelings… the greatest help was learning to be grateful. It clearly reduced my anxiety.”* (P68). In addition, one participant highlighted improvements in mindset and interpersonal skills *: “These modules helped me develop a better mindset and stronger interpersonal skills, which reduced my anxiety in daily life. This course gave me a healthier outlook. I worry less when facing setbacks and understand myself better. I’m more patient with others, and my life feels more peaceful—my anxiety has definitely reduced.”* (P65). Overall, 50% of participants cited self-affirmation as key in managing anxiety, and 40% emphasized gratitude as a way to reduce anxiety by fostering positive emotions.

Although the three primary themes—Increase in Happiness, Reduction in Depression, and Reduction in Anxiety—were most prominent in the qualitative dataset, the analysis also revealed neutral, mixed, and critical perspectives. Approximately 15% of students expressed neutral reflections, noting that the course did not noticeably change their well-being but still provided useful knowledge (e.g., “ *The course was interesting, but I cannot say it changed my happiness or mood much* .” P52). Around 12% of participants voiced mixed responses, indicating partial benefits combined with ongoing challenges (e.g., “ *Some modules made me feel calmer, but my anxiety still comes back during exams* .” P87). In addition, about 8% of students raised critical reflections, reporting discomfort or skepticism regarding certain modules. For example, several noted that relationship-focused content sometimes heightened pressure (“ *Discussions about family relationships made me feel more stressed because my situation is complicated* .” P41), while others questioned the long-term sustainability of course practices (“ *Gratitude journaling worked at first, but I stopped doing it after the course ended* .” P29). These perspectives were carefully considered during coding and theme development to avoid confirmatory bias. The presence of neutral, mixed, and critical reflections underscores the diversity of student experiences. Their inclusion during the analytic process helped refine coding decisions, ensure proportional representation, and confirm that the primary domains were adequately illustrated by student accounts rather than imposed solely by course objectives. In addition, it is noteworthy that student accounts referencing reductions in depression were more frequent and detailed than those addressing anxiety or happiness. This uneven distribution of qualitative material may reflect the relative salience of depressive symptoms in students’ lived experiences, and suggests that the course’s perceived impact was especially pronounced in this domain.

## 4 Discussion

This study evaluated the impact of an integrated KIPP and Positive Psychology course on university students’ psychological well-being in China, using a mixed-methods design that included pre-post quantitative analysis, fsQCA, and qualitative interviews. The findings offer valuable insights into how strength-based education—when culturally adapted and structurally anchored—can enhance students’ happiness while reducing symptoms of depression and anxiety.

First, to answer RQ1 and support H1a–H1c, paired-sample t-tests demonstrated statistically significant changes: happiness increased, depression decreased, and anxiety decreased. The effect sizes ranged from small to moderate, which is consistent with findings typically observed in educational well-being interventions. These results align with recent systematic reviews and meta-analyses showing that multi-component positive psychology interventions generally yield small-to-moderate improvements in well-being and reductions in distress among university students (Hendriks et al., 2019; Hobbs et al., 2022). The qualitative data further elaborated on the research questions and hypotheses of the study. For happiness, the statistical results showed a small but significant increase, which was further corroborated by the qualitative findings. Specifically, students reported increased happiness through enhanced emotional regulation, self-awareness, and gratitude, as captured in qualitative interviews. For example, one student shared, “ *I became more confident and positive, which significantly increased my happiness* ” (P34), which aligned with the quantitative finding of a moderate increase in happiness scores. This convergence of results across both datasets underscores the validity of the course’s impact on increasing happiness.

Similarly, for depression, the paired-sample t-test demonstrated a significant reduction in depressive symptoms, which was mirrored by the qualitative data. Students emphasized the role of the course in alleviating depression through strategies like emotional regulation, cognitive reframing, and meaning-making. For instance, one student noted, “ *I realized that life is not always joyful, but overcoming difficulties makes us stronger* ” (P9), aligning with the quantitative finding of a moderate decrease in depression scores. These qualitative accounts help deepen the understanding of how the course were associated with this reduction, with emotional resilience and social connections playing pivotal roles. Anxiety reduction, as shown by the paired-sample t-test result, was another key outcome of the course. Students frequently cited the importance of self-acceptance and emotional stability in managing anxiety. *“Practicing gratitude helped me shift focus from worries to positive feelings… the greatest help was learning to be grateful. It clearly reduced my anxiety.”* (P68). This qualitative insight complements the quantitative results, which indicated a significant decrease in anxiety scores, highlighting the role of both individual and social factors in alleviating anxiety.

Moreover, to address RQ2 and RQ3, the fsQCA results provided deeper insights into the specific course modules and configurations that are associated with improved psychological well-being. These configurational findings were both complemented and nuanced by insights derived from students’ qualitative reflections. The fsQCA findings showed that no single module was necessary for the observed improvements in psychological well-being. Instead, multiple combinations of modules functioned synergistically, reinforcing the value of adopting a systems-oriented perspective. These quantitative patterns were largely reflected in students’ subjective experiences, as captured through qualitative interview data. For example, both fsQCA and qualitative data pointed to the combination of “Discovering Strengths, Self-Regulation and Balance” and “Building Resilience” modules as particularly effective in increasing happiness. Students reported gaining enhanced self-awareness, confidence, and emotional regulation skills that allowed them to adopt more optimistic mindsets and reframe difficulties in adaptive ways. One student noted, “This course gave me a structured path for personal growth and helped me live a healthier, happier life” (P7), echoing the identified sufficiency of this configuration in the fsQCA analysis.

Similarly, fsQCA identified several pathways that effectively reduced depression, notably those involving Building Resilience, Finding Meaning and Joy, and Integrating Strengths into Daily Life. Students’ reflections corroborated this, with many expressing that the course helped them manage low moods by teaching them how to cultivate gratitude, reinterpret setbacks, and derive meaning from adversity. “I realized that life is not always joyful, but overcoming difficulties makes us stronger,” one student shared (P9), highlighting the existential and emotional coping mechanisms that aligned with the fsQCA pathways.

In terms of anxiety reduction, both analytic methods highlighted the importance of Integrating Strengths into Daily Life. FsQCA suggested that it could serve as a stand-alone sufficient condition for anxiety reduction. Qualitative reports echoed this, with students citing how applying learned character strengths in daily routines helped them feel more grounded, calm, and capable of navigating stress. For instance, one student stated, “Practicing gratitude helped me shift focus from worries to positive feelings… the greatest help was learning to be grateful. It clearly reduced my anxiety.” (P68).

However, it is worth noting that fsQCA identified a configuration where students who received “Strengthening Relationships” and “Integrating Strengths into Daily Life” without the support of more introspective modules (such as “Discovering Strengths” or “Building Resilience”) tended to report lower happiness levels. Some qualitative comments hinted at possible emotional strain from relational topics. For instance, a few students mentioned that content related to romantic or family relationships—central themes in the “Strengthening Relationships” module—was associated with feelings of discomfort or pressure. These students reported that their existing interpersonal difficulties were not always adequately addressed, and that expectations to “improve” relationships were experienced as an additional source of stress. This suggests that relational modules, while generally beneficial, may require greater scaffolding or emotional readiness to prevent unintended emotional burden—an important nuance not captured in fsQCA but visible through qualitative nuance. One possible explanation is that university students may experience heightened pressure when addressing relationship-building topics, such as romantic relationships or social interactions, which are central to the Strengthening Relationships module. Future research could further explore this dynamic to better understand how relationship-focused content impacts student well-being and stress levels.

The findings of the study are consistent with earlier work showing that positive psychology interventions enhance well-being and reduce psychological distress (Peterson and Seligman, 2004; Quinlan et al., 2019). Beyond this foundational evidence, more recent systematic reviews and meta-analyses underscore both the effectiveness and the cultural contingencies of positive psychology interventions. Multi-component positive psychology interventions are generally effective across populations, but outcomes are moderated by cultural and contextual factors, underscoring the importance of adaptation beyond Western contexts (Hendriks et al., 2019; Hendriks et al., 2020; van Agteren et al., 2021). In higher education specifically, positive psychology courses have been shown to significantly improve happiness, reduce depression and anxiety, and strengthen resilience among university students. Tarrats-Pons et al. (2025), for example, reported that positive psychology interventions alleviated depression and enhanced optimism among Spanish undergraduates. Similarly, Hobbs et al. (2022) reviewed 27 global studies and found that 85% reported positive outcomes, including increased life satisfaction and happiness, though many studies faced methodological limitations. Together, this literature positions positive psychology interventions as globally promising tools for promoting student well-being, while also highlighting the necessity of cultural tailoring to align with local values and practices.

At the same time, scholars have raised concerns about cultural transferability and contextual fit. Lambert et al. (2023), for instance, caution that positive psychology interventions, while beneficial, may risk promoting a one-size-fits-all model of well-being that does not always reflect the lived realities of students in non-Western contexts. Our qualitative findings provide partial support for this concern. Several students reinterpreted course content through culturally specific values, such as relational harmony and emotional moderation, and expressed ambivalence toward modules focused on interpersonal change. In particular, 15% of students reported neutral experiences, suggesting that although the course was informative, its impact was uneven and may not have reached students whose baseline well-being was less affected. Another 12% described mixed outcomes, highlighting challenges in sustaining benefits outside the classroom. This underscores the potential value of follow-up strategies—such as booster sessions, digital tools, or reflective assignments—to reinforce skills and promote long-term change. Meanwhile, 8% voiced critical reflections, particularly regarding the relational modules. Sensitive topics such as family or romantic relationships, while important, sometimes generated additional stress or discomfort. These concerns point to the need for greater scaffolding, optional participation, or supplementary support resources (e.g., small-group guidance or counseling referrals) to ensure students feel both supported and respected when engaging with such content. Addressing these neutral, mixed, and critical perspectives not only mitigates unintended stress but also enhances the inclusivity and sustainability of the program. By integrating culturally sensitive adjustments and responsive scaffolding, the course can evolve beyond a standardized model toward a strengths-based approach that is both adaptable and more deeply aligned with the diverse needs of Chinese university students.

Despite the promising findings, this study has several limitations. First, the research was conducted at a single university in southeastern China, which may constrain the generalizability of the results to other higher education contexts. Replicating this study across diverse institutional and regional settings would enhance the external validity of the findings. Second, although the pre-post comparisons indicate significant short-term improvements in psychological well-being, the durability of these effects over time remains unknown. Future longitudinal studies should include follow-up assessments to evaluate the long-term impact of the course. Third, while the removal of one item from each of the happiness and depression scales was informed by semantic ambiguity and cultural inappropriateness based on participant feedback, this modification may limit direct comparability with prior studies using the full standardized scales. Future research should retain all original items to facilitate cross-cultural validation and ensure consistency with existing literature. Fourth, the absence of a control group limits the ability to attribute observed changes solely to the intervention. Future studies could adopt a randomized design to more rigorously assess causality. However, the combination of pre-post comparisons, fsQCA configurational analysis, and student interviews offers triangulated evidence that strengthens internal validity despite this limitation. Fifth, while all survey responses were confidential, the lack of complete anonymity in data collection may have introduced some degree of social desirability bias. To mitigate this, participants were reminded that their responses would not affect their grades or be shared with instructors. Besides, while fsQCA enabled the identification of multiple configurations contributing to well-being outcomes, this method also carries specific limitations. For example, fsQCA results are sensitive to the calibration thresholds chosen during data preparation, which introduced a degree of subjectivity in translating continuous variables into fuzzy sets. Although we followed established guidelines and used both empirical and theoretical justifications for our calibration anchors, the potential for interpretive bias remains. Future research may benefit from combining fsQCA with longitudinal or hybrid-comparative designs—such as panel studies or mixed-methods quasi-experiments—to triangulate findings. Finally, given that interview questions explicitly referenced happiness, depression, and anxiety, qualitative evidence may overweight those domains. While the use of directed content analysis usefully complements the quantitative outcomes, it inherently constrains the scope of findings by focusing on pre-specified domains and does not capture broader or unexpected aspects of student experience. We partially mitigated this by training interviewers to probe for unexpected, null, or negative effects, quantifying neutral/mixed/critical content, and reporting counter-examples. Future work should incorporate broader life-domain prompts (e.g., day-in-the-life narratives), blinding interviewers to hypotheses, and separating interview timing from course completion to further reduce demand characteristics.

This study adds to the growing literature on integrated positive psychology interventions in higher education by explicitly embedding the KIPP character framework into a Chinese university context. Unlike programs focusing solely on cognitive or emotional skills, this course emphasized applied character development across emotional, social, and existential domains, aligning with calls for holistic and culturally adapted well-being education. Moreover, by adopting a mixed-methods framework that combines quantitative, configurational, and qualitative evidence, our study extends beyond outcome evaluation to uncover the specific mechanisms and student experiences that drive psychological change. Our findings carry practical implications for curriculum design and mental health promotion in higher education. First, modules that target self-regulation, resilience, and meaning-making—especially when delivered in an integrated fashion—should be prioritized. Second, educators should recognize that interpersonal modules may be double-edged: while they offer opportunities for emotional connection, they may also heighten stress for students unprepared to navigate relational vulnerability. Providing adequate scaffolding and optional engagement modes can help mitigate this issue. At the policy level, this study affirms the feasibility and potential benefits of embedding structured character education into general education courses. The global expansion of positive psychology education—documented in Australia, the U. S., and other nations (Allen et al., 2017; Seligman and Adler, 2018)—finds resonance in our findings, especially in light of the unique challenges faced by Chinese university students, such as academic stress, perfectionism, and stigma around mental health help-seeking. As mental well-being becomes an increasingly recognized pillar of educational success, programs like “Building a Flourishing Life” offer evidence-informed strategies to promote flourishing in youth populations. Additionally, the findings highlight the necessity of an approach that integrates multiple character strengths rather than relying on isolated interventions. Beyond curriculum design, these findings have broader implications for mental health policies in higher education. Universities should consider incorporating strength-based interventions into counseling and support services, providing students with structured opportunities to cultivate resilience and well-being. Given the significant reductions in depression and anxiety observed in this study, there is strong justification for scaling up such interventions to reach a broader student population.

## Data Availability

The raw data supporting the conclusions of this article will be made available by the authors, without undue reservation.
