# Supplementary material for: Evaluating the impact of an integrated KIPP and positive psychology course on university students’ psychological well-being in China: a mixed-methods study
Source: Front Psychol. 2025 Oct 6;16:1599216. doi: 10.3389/fpsyg.2025.1599216 (PMC12535991; doi:10.3389/fpsyg.2025.1599216)
Supplement: Supplementary file 1 [file Table_1.pdf]

Supplementary Table 1. Mapping of course modules, sample activities, and aligned KIPP/VIA character strengths implemented in the intervention (N = 116).

Course Module	Sample Activities	Corresponding KIPP/VIA Strengths
Strengthening Relationships	Gratitude letters, active listening role-play, empathy mapping	Kindness, Love, Social Intelligence, Empathy, Perspective
Discovering Strengths, Self-Regulation & Balance	VIA strength reflection, self-regulation journal, emotion log	Self-regulation, Perseverance, Emotional Awareness, Zest, Perspective
Building Resilience	Growth mindset storytelling, coping skills rehearsal, strength-spotting	Grit, Optimism, Courage, Hope, Perspective
Finding Meaning and Joy (Transcendence)	Gratitude journaling, meaning-in-life exercises, mindfulness practice	Gratitude, Appreciation of Beauty, Hope, Spirituality
Integrating Strengths into Daily Life	Strength application plan, habit tracker, reflective presentations	Zest, Self-Awareness, Grit, Optimism, Self-Compassion

Each course module (e.g., Strengthening Relationships, Discovering Strengths/Self-Regulation & Balance, Building Resilience, Finding Meaning and Joy/Transcendence, Integrating Strengths into Daily Life) is illustrated with representative activities delivered during the course (such as gratitude letters, self-regulation journals, coping skills rehearsal, mindfulness practice, and strength application plans). Corresponding KIPP/VIA character strengths (e.g., kindness, love, perseverance, grit, optimism, spirituality) are listed to demonstrate the integration of character education and positive psychology principles.

Supplementary Table 2 Descriptive calibration values for fsQCA across outcomes (happiness, depression, anxiety).

	Happiness	Depression	Anxiety
Minimum	0	1	0
Maximum	10	10	10
Mean	7.8	7.6	8.1
Calibration			
5th percentile	5	3	1
50th percentile	8	8	9
95th percentile	10	10	10

Reported values include minimum, maximum, mean, and calibration thresholds (5th, 50th, 95th percentiles).Main descriptions and calibration values.

Supplementary Table 3. Necessary conditions for high and low levels of happiness identified using fsQCA.

	High levels of happiness		Low levels of happiness	
Conditions	Consistency	Coverage	Consistency	Coverage
Strengthening Relationships	0.51	0.49	0.56	0.51
~Strengthening Relationships	0.49	0.55	0.44	0.46
Discovering Strengths, Self-Regulation and Balance	0.37	0.60	0.26	0.40
~Discovering Strengths, Self-Regulation and Balance	0.63	0.48	0.74	0.52
Building Resilience	0.76	0.56	0.63	0.44
~Building Resilience	0.24	0.41	0.37	0.59
Finding Meaning and Joy (Transcendence)	0.47	0.48	0.55	0.52
~Finding Meaning and Joy (Transcendence)	0.53	0.55	0.45	0.45
Integrating Strengths into Daily Life	0.38	0.534	0.36	0.47
~Integrating Strengths into Daily Life	0.62	0.516	0.64	0.49

Consistency and coverage values are reported for each course module (Strengthening Relationships, Discovering Strengths/Self-Regulation & Balance, Building Resilience, Finding Meaning and Joy/Transcendence, Integrating Strengths into Daily Life). Presence (X) and absence (~X) of conditions are included. Consistency indicates the degree to which a condition is necessary, while coverage indicates the empirical relevance of the condition.

Supplementary Table 4. Necessary conditions for high and low levels of depression based on fsQCA.

	High levels of depression		Low levels of depression	
Conditions	Consistency	Coverage	Consistency	Coverage
Strengthening Relationships	0.52	0.53	0.55	0.47
~Strengthening Relationships	0.48	0.56	0.45	0.44
Discovering Strengths, Self-Regulation and Balance	0.32	0.55	0.32	0.45
~Discovering Strengths, Self-Regulation and Balance	0.68	0.54	0.68	0.46
Building Resilience	0.73	0.57	0.66	0.43
~Building Resilience	0.27	0.48	0.34	0.52
Finding Meaning and Joy (Transcendence)	0.54	0.57	0.48	0.43
~Finding Meaning and Joy (Transcendence)	0.46	0.51	0.52	0.49
Integrating Strengths into Daily Life	0.40	0.59	0.33	0.41
~Integrating Strengths into Daily Life	0.60	0.52	0.67	0.48

Consistency and coverage values are reported for the presence and absence of each course module. Higher consistency suggests a condition is necessary for the outcome, whereas coverage reflects the proportion of cases explained by that condition.

Supplementary Table 5. Necessary conditions for high and low levels of anxiety derived from fsQCA.

	High levels of anxiety		Low levels of anxiety	
Conditions	Consistency	Coverage	Consistency	Coverage
Strengthening Relationships	0.57	0.60	0.49	0.40
~Strengthening Relationships	0.43	0.52	0.51	0.48
Discovering Strengths, Self-Regulation and Balance	0.33	0.58	0.30	0.42
~Discovering Strengths, Self-Regulation and Balance	0.67	0.55	0.70	0.45
Building Resilience	0.67	0.54	0.73	0.46
~Building Resilience	0.33	0.62	0.27	0.38
Finding Meaning and Joy (Transcendence)	0.54	0.60	0.47	0.40
~Finding Meaning and Joy (Transcendence)	0.46	0.53	0.53	0.47
Integrating Strengths into Daily Life	0.40	0.60	0.34	0.40
~Integrating Strengths into Daily Life	0.60	0.54	0.66	0.46

Consistency and coverage values are reported for each course module in its presence and absence form. Results identify conditions that are necessary for experiencing higher or lower anxiety levels among students.

Supplementary Table 6. Sensitivity analyses of calibration thresholds for outcome variables (happiness, depression, anxiety).

	Happiness	Depression	Anxiety
Minimum	0	1	0
Maximum	10	10	10
Mean	7.8	7.6	8.1
Calibration			
10th percentile	5	6	5.5
45th percentile	8	9	8
90th percentile	10	10	10

Reported values include observed minimum, maximum, and mean scores, as well as calibration cutoffs (10th, 45th, and 90th percentiles). These thresholds were applied to transform raw data into fuzzy-set membership scores.

Supplementary Table 7. Sensitivity analysis of configurations thatare associated with happinessusing fsQCA.

Configuration	Solution High	Solution Low
	1	2
Strengthening Relationships	○	●
Discovering Strengths, Self-Regulation and Balance	●	○
Building Resilience	●	
Finding Meaning and Joy (Transcendence)	○	○
Integrating Strengths into Daily Life		●
Consistency	0.85	0.85
Raw coverage	0.10	0.04
Unique coverage	0.10	0.04
Solution consistency	0.85	0.85
Solution coverage	0.10	0.04

● = presence of a core condition; ■ =presence of a peripheral condition; ○ = negation of a core condition; □ = negation of a peripheral condition; Blank spaces indicate that a condition is not relevant in a configuration.

Supplementary Table 8. Sensitivity analysis of configurations that are associated with depression.

Configuration	Solution High			Solution Low	
	1	2	3	4	5
Strengthening Relationships	●	○	●	●	●
Discovering Strengths, Self-Regulation and Balance	○	●	○	●	○
Building Resilience	●	●	○	○	○
Finding Meaning and Joy (Transcendence)	●	○	●	●	○
Integrating Strengths into Daily Life	○	●	●	●	●
Consistency	0.82	0.80	0.80	0.95	0.88
Raw coverage	0.03	0.04	0.04	0.02	0.02
Unique coverage	0.03	0.04	0.04	0.02	0.02
Solution consistency	0.80			0.91	
Solution coverage	0.11			0.03	

● = presence of a core condition; ■ =presence of a peripheral condition; ○ = negation of a core condition; □ = negation of a peripheral condition; Blank spaces indicate that a condition is not relevant in a configuration.

Supplementary Table 9. Sensitivity analysis of configurations that are associated with anxiety.

Configuration	Solution High	
	1	2
Strengthening Relationships	○	●
Discovering Strengths, Self-Regulation and Balance	○	○
Building Resilience	○	●
Finding Meaning and Joy (Transcendence)	○	●
Integrating Strengths into Daily Life	●	○
Consistency	0.95	0.95
Raw coverage	0.02	0.03
Unique coverage	0.02	0.03
Solution consistency	0.95	
Solution coverage	0.05	

● = presence of a core condition; ■ =presence of a peripheral condition; ○ = negation of a core condition; □ = negation of a peripheral condition; Blank spaces indicate that a condition is not relevant in a configuration.
